# Multilevel Space-Time Aggregation for Bright Field Cell Microscopy Segmentation and Tracking

**DOI:** 10.1155/2010/582760

**Published:** 2010-04-27

**Authors:** Tiffany Inglis, Hans De Sterck, Geoffrey Sanders, Haig Djambazian, Robert Sladek, Saravanan Sundararajan, Thomas J. Hudson

**Affiliations:** ^1^Centre for Computational Mathematics in Industry and Commerce, University of Waterloo, Waterloo, ON, Canada N2L 3G1; ^2^Department of Applied Mathematics, University of Waterloo, Waterloo, ON, Canada N2L 3G1; ^3^Department of Applied Mathematics, University of Colorado at Boulder, Boulder, CO 80309, USA; ^4^McGill University and Genome Quebec Innovation Centre, Montreal, QC, Canada H3A 1A4; ^5^Ontario Institute for Cancer Research, Toronto, ON, Canada M5G 0A3

## Abstract

A multilevel aggregation method is applied to the problem of segmenting live cell bright field microscope images. The method employed is a variant of the so-called “Segmentation by Weighted Aggregation” technique, which itself is based on Algebraic Multigrid methods. The variant of the method used is described in detail, and it is explained how it is tailored to the application at hand. In particular, a new scale-invariant “saliency measure” is proposed for deciding when aggregates of pixels constitute salient segments that should not be grouped further. It is shown how segmentation based on multilevel intensity similarity alone does not lead to satisfactory results for bright field cells. However, the addition of multilevel intensity variance (as a measure of texture) to the feature vector of each aggregate leads to correct cell segmentation. Preliminary results are presented for applying the multilevel aggregation algorithm in space time to temporal sequences of microscope images, with the goal of obtaining space-time segments (“object tunnels”) that track individual cells. The advantages and drawbacks of the space-time aggregation approach for segmentation and tracking of live cells in sequences of bright field microscope images are presented, along with a discussion on how this approach may be used in the future work as a building block in a complete and robust segmentation and tracking system.

## 1. Introduction

There is extensive current interest in the high-content, high-throughput screening of live cell populations, and the experimental techniques being developed for this purpose are leading to important biological insights with applications to new clinical therapies [1–4]. Due to the large amount of data generated by these experiments, automated data processing systems for segmentation and tracking of live cells in sequences of microscope images are a virtual necessity. Several comprehensive systems for segmentation and tracking have recently been described in the literature (see, e.g., [5, 6] and references therein). In most existing approaches, cells are segmented using advanced level set, active contour or watershed methods, or ingenious combinations of them [5–7]. In this paper, we investigate the use of a multilevel aggregation algorithm as an alternative method for segmenting live cell bright field microscope images. We use a variant of the so-called “Segmentation by Weighted Aggregation” (SWA) technique [8, 9]. Our algorithmic contribution is a new scale invariant “saliency measure” for this algorithm to decide when aggregates of pixels constitute salient segments that should not be grouped further. We investigate the performance of the multilevel aggregation algorithm for bright field cell segmentation in images and sequences of images, and discuss its advantages and drawbacks compared to other methods as a possible building block in comprehensive automatic cell segmentation and tracking systems.

### 1.1. Problem Description

**Figure 1:**
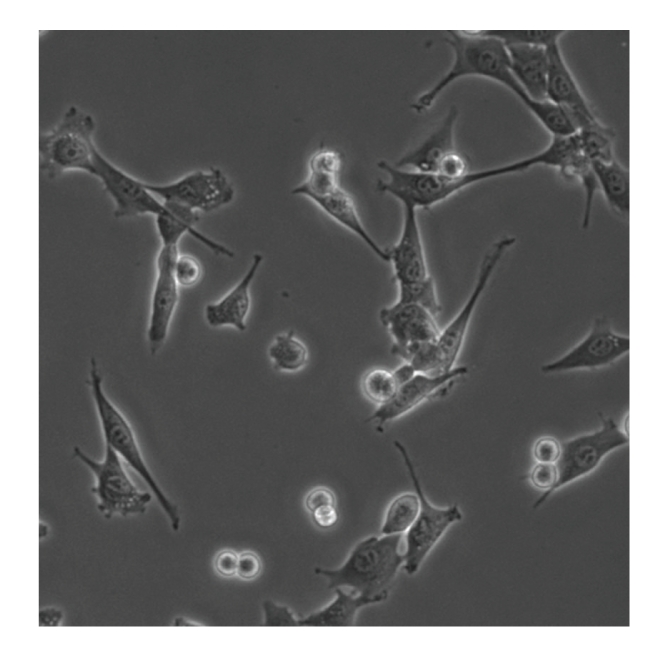
Bright field microscope image with approximately two dozen cells on a grey background. Some interior structure can be discerned in cells (including the cell membrane, the dark grey cytoplasm, and the lighter cell nucleus with dark nucleoli inside). Cells that are close to division appear as bright, nearly circular shapes. Some cells are touching, and some cell parts overlap. Our goal is to obtain separate segments for each cell in the image.

Figure 1 shows a microscope image with approximately two dozen mouse C2C12 myoblast cells on a dark grey background. C2C12 myoblasts (ATCC CRL-1772) are muscle cell precursors, which have varied shape and size as well as good motility in vitro. In tissue culture, they will grow to a fully confluent monolayer with well-separated cell nuclei and a small amount of overlapping cytoplasm between adjacent cells.

Our goal is to obtain separate segments for each cell in images like Figure 1. It can be seen immediately from Figure 1 that this is a difficult task. Two major challenges are, first, that the cells appear in two distinct basic shapes (cells that are close to division show as bright circular shapes, while regular cells have more stretched shapes in which the cytoplasm and nucleus can be discerned), and, second, that cells may be touching or overlapping. While our ultimate goal is to automatically segment sequences of complex images like Figure 1, we start out in this paper with segmentation of simplified cases (isolated cells and small groups of cells).

### 1.2. Approach and Algorithm

We use a variant of the Segmentation by Weighted Aggregation technique [8, 9] to segment the microscope images, which itself is based on Algebraic Multigrid (AMG) methods for solving linear systems of equations [10, 11]. The SWA method attempts to segment the image into salient (or “prominent”) groups of pixels by using a multilevel aggregation procedure that groups blocks of pixels at various scales, based on multiscale feature vectors for the pixel blocks. The SWA method has more recently also been used as the basis of a more general multilevel clustering algorithm [12, 13]. The variant of the method that we use is described in detail below, and it is explained how it is tailored to the application at hand. Our algorithmic contribution is a new scale invariant “saliency measure” for deciding when aggregates of pixels constitute salient segments that should not be grouped further. Our new saliency measure is a variant of the saliency measure employed in [8, 9] that takes scaling into account. Furthermore, we use the so-called “first pass” of the standard AMG graph coarsening algorithm [10, 11] to coarsen the pixel graph, rather than the direct aggregation methods that are used in SWA algorithms described in the literature [8, 9, 12, 14]. We test our approach on parts of real microscope images, and show how segmentation based on multilevel intensity similarity alone does not lead to satisfactory results. However, the addition of multilevel variance of mean intensities (as a measure of texture) to the feature vector of each aggregate, as in [9, 14], leads to correct cell segmentation.

In the second part of the paper, preliminary results are presented for applying the multilevel aggregation algorithm in space time to temporal sequences of microscope images, with the goal of obtaining space time segments (object tunnels) that track individual cells. This parallels previous research results on space time segmentation using level set methods [15, 16] and on comprehensive segmentation and tracking systems for sequences of cell images [5, 6], but space time segmentation using SWA has, to our knowledge, not been investigated in the literature yet. The ultimate goal of this space time segmentation is to determine a space time segment (object tunnel) for each individual cell, taking into account cell divisions. Ideally, when a cell divides, the segment of the mother cell should terminate and cell segments should start for the two daughter cells. In this sense, the desired outcome of our space time segmentation is more complicated than for related problems such as cerebral vasculature segmentation in CT scans, for which three-dimensional level set algorithms have been developed [17]. Also, the full space time segmentation problem is complicated since cells change shape when they divide and cells may touch and overlap. In this paper, we present preliminary results on our experience with extending the spatial SWA segmentation algorithm to space time segmentation of sequences of images, for simplified cases of isolated cells and pairs of cells. We identify problems that remain and suggest possible extensions of the algorithm that may handle them, and we comment on the potential of the SWA segmentation method as a building block in comprehensive segmentation and tracking systems along the lines of [5, 6] and references therein.

While the general ideas and concepts of the SWA framework are described in several places [8, 9, 12, 14], most of the description in the literature is formulated in general terms, and not all algorithmic details are spelled out. In this paper, we want to present methods and results that are fully reproducible by the reader, and for this reason we provide a complete description of the version of the SWA algorithm we settled on. One property of the SWA algorithm is that it has a significant number of parameters that have to be chosen judiciously to obtain the desired segmentation. While this may be perceived as a potential drawback of the approach, it also opens opportunities to finetune the algorithm for the application class at hand. Indeed, the problem of segmenting an image without further specifications is often ill-posed. For example, a satellite image with roads, buildings, cars and people can be segmented at the level of the roads and buildings, or at the more detailed level of individual cars and people. Finetuning the SWA parameters allows the user to steer the segmentation in the desired direction. The question about how to choose the SWA parameters is not discussed much in the literature [8, 9, 12, 14], and in most cases specific parameters are not given for segmentation results that are presented as examples. Again in the interest of reproducibility, we make sure in this paper to include full details about the parameters we choose for each image or image sequence for which we give segmentation results, and we also formulate some general guidelines for choosing parameters.

### 1.3. Context

Automatic segmentation and tracking of live cells in bright field microscopy images is a difficult task [5, 18]. For this reason, fluorescence microscopy is often used in place of bright field microscopy to image live cells. In fluorescence microscopy, cells are made fluorescent by applying fluorescent dyes, or by making the cells artificially express fluorescent proteins. The strong fluorescence signals facilitate tracking [6], but may also have significant drawbacks. In many cases, the fluorescence introduced is toxic for the cells [19], and it may change cell behaviour. On the contrary, bright field microscopy is well suited for live cell studies as the imaging conditions can usually be chosen to minimize phototoxicity.

For example, the experiments reported in [20] show how the shape of a cell can be linked to genetic factors, in this case signaling proteins. In this study, the cells were fixed and permeabilized prior to phalloidin staining and, since the cells are no longer alive, it is impossible to examine the same cells at a later time. Killing the cells is often a necessary step when using stains. However, the image segments we obtain from our analysis can be used to categorize cell shapes while keeping the cells in culture. This ensures minimal interference with normal cell function through toxicity from photochemical effects and the high intensity illumination that is required to excite fluorescent probes.

In other types of experiments, fluorescent markers are used to measure concentrations of certain specific proteins that are under study. A limited number of fluorescent marker channels with nonoverlapping spectra (typically up to three) are used in combination. If one or two of these channels are used solely for tracking purposes, the number of channels available for measuring protein concentrations is reduced, which may be a significant limitation.

For these reasons, it can be an advantage if methods can be derived that manage to track cells based on bright field images [5].

In addition, bright field images typically contain many features of interest, and accurate segmentation methods for bright field images are intrinsically useful since they allow the study of cell morphology and internal cell structure, and their dynamics. For example, the morphology of a cell can be indicative of cell health or can indicate various stages of pathology [20]. Also, accurate bright field segmentation of cells and cell organelles may allow for more precise integration of fluorescence signals over relevant parts of precisely identified cells, enhancing the accuracy of protein expression level measurements. Finally, it should also be noted that the contrast of bright field images is often enhanced by the use of phase contrast microscopy. The images discussed in this paper were obtained with this technique. This allows us to see detailed structure within cells on Figure 1 (including cell nuclei and nucleoli), but it also generates bright halos around cells which may complicate segmentation since they do not uniformly enclose cells.

The microscope images used in this paper (including Figure 1) were obtained as follows. Cells were plated at a low density in glass chamber slides and imaged at 20X using a phase contrast objective (Nikon TEU 2000 with CFI Plan Fluor ELWD 20X) and a cooled CCD camera (Hamamatsu C9100-12). Each image contains 512 by 512 pixels, with a resolution of 0.8 *μ*m/pixel and 14 effective bits per pixel. Unless otherwise noted, all cell culture reagents were purchased from Invitrogen. C2C12 myoblasts were cultivated in growth medium (Dulbecco's Modified Eagle Medium supplemented with 20% fetal bovine serum (Sigma), 2 mM glutamine, 50 units penicillin and 50 *μ*g/mL streptomycin in 10 cm cell culture plates (Corning). Cells were treated with 1.5 mL of 0.05% Trypsin-EDTA for 5 minutes, manually dispersed by pipetting and diluted in growth medium to 50,000 cells/mL. Subsequently, 3 mL of this suspension was added to one Lab-Tek chambered coverglass well (Nunc) and cells were allowed to settle undisturbed at 37°C and 10% CO_2_ for 16 hours. The slide was then placed on the motorized stage of a Nikon TEU 2000 equipped with a Solent environmental chamber where the cells were kept at the same CO_2_ and temperature conditions as before. The microscope images were acquired at five-minute intervals during 24 hours.

### 1.4. Organization of the Paper

The rest of this paper is structured as follows. In Section 2, we give a brief description of the SWA algorithm and introduce a new scale invariant saliency measure. In Section 3 we investigate how the algorithm performs for segmenting bright field cell images. In Section 4 we explain how the approach is extended to segmentation in space time in a straightforward way, and investigate the performance of space time segmentation for simple sequences of real cell images. Computational cost and scalability of the SWA algorithm are discussed in Section 5. Section 6 discusses conclusions and future work. Finally, Appendix A gives a pseudocode-style step-by-step description of our algorithmic implementation.

## 2. Algorithm Description

In this section, we describe the multilevel segmentation algorithm that we employ in this paper. We start with an overview of the basic SWA algorithm from [8, 9, 12–14, 21], followed by a subsection on some specific more detailed aspects of the algorithm. In the third subsection, we propose and discuss a new scale invariant saliency measure for the SWA algorithm. For a complete description of the algorithm we use, we refer the reader to Appendix A, which provides an actual pseudocode-style step-by-step description of our implementation.

### 2.1. Overview of Algorithm

**Figure 2:**
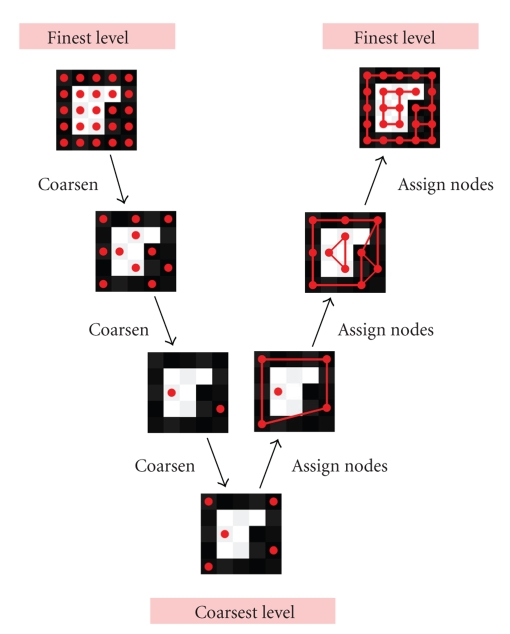
Schematic representation of the SWA algorithm.

Figure 2 shows a schematic representation of the SWA algorithm. A high-level description of the SWA algorithm proceeds as follows. In the first, top-down phase of the algorithm, pixels are recursively grouped into increasingly large overlapping blocks. At any level in the process, blocks that are sufficiently different from their neighbours are identified as salient segments. The top-down phase ends when all remaining blocks have become salient. In the second, bottom-up phase of the algorithm, overlapping blocks that were identified as salient are recursively sharpened, until all fine-level pixels are assigned to a unique segment at the top level.

We now give a more detailed description of the algorithm.

The problem of image segmentation can be viewed in terms of segmentation of a weighted undirected graph, with each node of the graph representing a pixel, and each edge corresponding to a link between neighbouring nodes, weighted by the similarity in intensity between the two neighbouring pixels. The SWA algorithm starts with this weighted graph on the finest level (which we call level 1) and forms a weighted graph of reduced size on level 2, with the nodes of the level-2 graph representing overlapping blocks of level-1 nodes. Each level-2 block is formed around a level-1 seed point, which is called a *C*-point (short for coarse point) on level 1. Nodes on level 1 that are not chosen as *C*-points are called *F*-points (short for fine points). The overlapping blocks are chosen such that they group neighbouring nodes that have similar intensities. This graph coarsening can be done in a variety of ways, and the particular approach we employ is explained in the next subsection and in Appendix A.3. This graph coarsening process is then repeated on level *r*, *r* = 2,3, 4,…, to obtain the coarse-level graph on level *r* + 1. The left branch of the diagram in Figure 2 shows for an example image how the nodes (shown as red dots) are coarsened during the first, top-down phase of the algorithm. (For each coarse-level overlapping block, one finest-level node corresponding to the *C*-point of the block on the previous level is shown as a representative.)

In more specific terms, we start from the pixel graph of the original image, with the intensities of the pixels stored in level-1 intensity vector *I* ^[1]^. (We scale each input image such that the maximum intensity value equals 1.) Pixels that are neighbours in the horizontal or vertical directions are connected by weighted edges in the graph, with edge weights *A*_*ij*_^[1]^ defined using an exponential function of the intensity difference between the pixels they connect:

(1) $$\begin{array}{c} A_{ij}^{\left[1\right]}=\{\begin{array}{cc} e^{-\alpha |I_{i}^{\left[1\right]}-I_{j}^{\left[1\right]}|} & \text{if}\;\;i,j\;\;\text{are}\;\;\text{neighbours},\\ 0, & \text{otherwise}, \end{array} \end{array}$$

with *α* ≥ 0 a user-defined parameter. *C*-points are then chosen for the level-1 graph and their indices are stored in vector *C* ^[1]^. An interpolation matrix from level 2 to level 1 is defined as

(2) $$\begin{array}{c} P_{ij}^{\left[1,2\right]}=\{\begin{array}{cc} 1 & \text{if}\;\;i\in C^{\left[1\right]},\;\;i=C_{j}^{\left[1\right]},\\ 0 & \text{if}\;\;i\in C^{\left[1\right]},\;\;i\neq C_{j}^{\left[1\right]},\\ \frac{A_{iC_{j}^{\left[1\right]}}^{\left[1\right]}}{\sum_{k\in C^{\left[1\right]}}^{}A_{ik}^{\left[1\right]}} & \text{if}\;\;i\notin C^{\left[1\right]}. \end{array} \end{array}$$

Here we use the shorthand notation *i* ∈ *C* ^[1]^ to mean that *i* = *C*_*j*_^[1]^ for some *j*. Each column *j* of *P* ^[1,2]^ represents a level-2 node, and element *P*_*ij*_^[1,2]^ indicates which fraction of level-1 node *i* belongs to level-2 block *j*. (The rows of *P* ^[1,2]^ sum to one.) The edge weights between nodes of the level-2 graph are calculated by an averaging process known as Galerkin coarsening [11]:

(3) $$\begin{array}{c} A^{\left[2\right]}=P^{[1,2]T}A^{\left[1\right]}P^{\left[1,2\right]}. \end{array}$$

In a similar way, interpolation operators *P* ^[*r*,*r*+1]^ are derived between the other recursive levels, and coarse-level graph weights are calculated by

(4) $$\begin{array}{c} A^{\left[r+1\right]}=P^{[r,r+1]T}A^{\left[r\right]}P^{\left[r,r+1\right]}. \end{array}$$

On each level, the blocks are tested for saliency: a salient (or “prominent”) block is a block that is sufficiently different from all the blocks it is connected to, as determined by a saliency measure. Once a block is designated salient, it is not allowed to merge with other blocks on coarser levels. (In our implementation, salient blocks are propagated to all coarser levels.) The coarsening process terminates at the level where all blocks have become salient, at which point we have found all segments in the image. In Figure 2, the bottommost image is at the coarsest level where coarsening stops because each of the two nodes represents a salient segment (the white and the black segment).

The right branch of the diagram in Figure 2 represents the second, bottom-up phase of the algorithm. Recall that the segments obtained on the coarsest level correspond to overlapping blocks on the finest level. It is important to retain overlapping blocks in the first phase of the algorithm, since a pixel that may appear to belong primarily to a certain block according to fine-level information, may be reclassified as belonging to a different block later when coarser-level information is taken into account. The goal of the second phase is to uniquely assign every finest-level pixel to one of the segments. The nodes on the second-to-coarsest level are first assigned to the segments obtained on the coarsest level according to the weights in the interpolation matrix, and this is repeated recursively for nodes on increasingly fine levels. In this way, the representation of the so-far overlapping segments is obtained on each recursive level. On each level, the overlap between segments is reduced by a sharpening procedure, with a sharpening threshold *d*_1_, which we normally take equal to 15%. In this sharpening procedure, nodes that belong for more than 85% to a segment are assigned for 100% to that segment, while nodes that belong for less than 15% to a segment are fully decoupled from that segment. Finally, on the finest level, every pixel is assigned to the segment to which it belongs the most.

The SWA algorithm is in many ways similar to AMG methods for solving linear systems of equations [10, 11], and the V-shaped process of Figure 2 is called a V-cycle in multigrid terminology. Contrary to the AMG V-cycle, where cycles are repeated to iteratively produce better approximations to linear equations, we perform the image segmentation V-cycle only once, giving the final segmentation.

**Figure 3:**
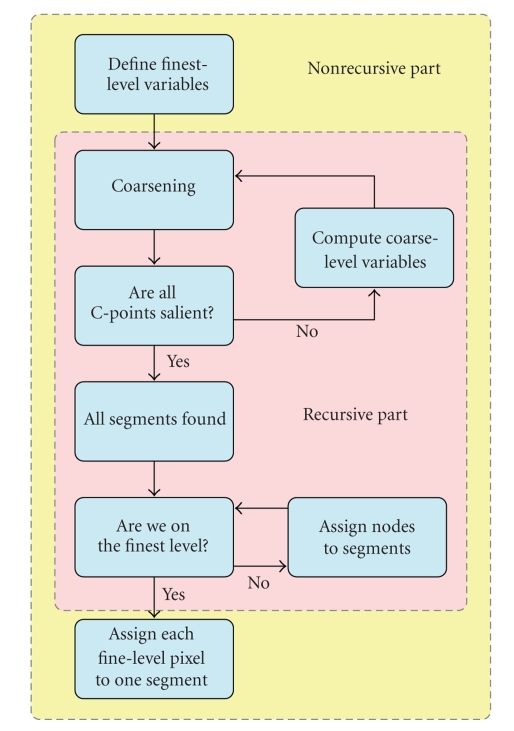
Flow chart for the algorithm with the recursive and nonrecursive parts.

Figure 3 shows a flow chart of the SWA algorithm. The core of the algorithm can be described recursively. The recursive part of the algorithm is preceded and followed by a nonrecursive part on the finest level. In the recursive part we consider two adjacent levels: level *r* (the current fine level) and level *r* + 1 (the current coarse level). On level *r*, we find a suitable subset of fine-level nodes (the *C*-points) that will form the seed points for the coarse-level blocks. Then, as long as we are not yet on the coarsest level (i.e., not all nodes are salient), we calculate the coarse-level graph weights from the fine-level weights, and coarsen again recursively. Once on the coarsest level, the recursive function returns the segments found, and performs a bottom-up process that ultimately leads to each pixel in the (finest-level) image being assigned to exactly one of the (initially overlapping) segments found on the coarsest level. The nonrecursive and recursive parts of the algorithm are described in detail in Appendices A.1 and A.2, respectively, and the coarsening algorithm is given in Appendix A.3. Calculation of the saliency measure to detect segments is discussed in Section 2.3.

### 2.2. Additional Algorithmic Elements

The following are further details and enhancements of the SWA algorithm.

- In order to coarsen the graph at the current level, we use the so-called first pass of the standard AMG coarsening algorithm [10, 11]. This algorithm coarsens the connectivity graph with weights *A*_*ij*_^[*r*]^ by first dividing the connections in sets of weak and strong connections based on their weights, and then approximately determining a subset of the nodes of the graph that is a maximal independent set in the subgraph formed by only retaining the strongly connected edges. See Appendix A.3 for a full description of the coarsening algorithm we employ. Strength parameter *θ* ∈ [0,1] (see (A.22)) is used as a threshold to determine strong connections in the coarsening algorithm. The nodes in the maximal independent set, which are selected such that they are not linked by strong connections, become the *C*-points on the current level. Nodes that have strong connections to many other nodes are more likely to be chosen as *C*-points. Note also that, on coarse level *r*, nodes that have already been designated salient on previous levels (see Section 2.3), automatically become *C*-points at the beginning of the coarsening on level *r*. Note that we use standard AMG coarsening [10, 11] to coarsen the graph, rather than the direct aggregation methods that are used in SWA algorithms described in the literature [8, 9, 12, 14]. We find the standard AMG coarsening a simple and effective graph coarsening algorithm that leads to good results, as confirmed by extensive experiments.
- Calculating *A* ^[*r*+1]^ by
  (5) $$\begin{array}{c} A^{\left[r+1\right]}=P^{[r,r+1]T}A^{\left[r\right]}P^{\left[r,r+1\right]} \end{array}$$
  gives the coupling weight between two blocks based on finer-level coupling weights across their shared boundary. However, it is beneficial to also consider the direct similarity between the blocks in terms of their average intensity. The average intensity of each block on level *r* + 1 can easily be calculated from the average intensity on level *r* by multiplication with a scaled interpolation matrix, see (A.11) and (A.10). The coupling weights can then be rescaled as follows to incorporate similarity in average intensity:
  (6) $$\begin{array}{c} A_{ij}^{\left[r+1\right]}⟵A_{ij}^{\left[r+1\right]}e^{-\tilde{\alpha }|I_{i}^{\left[r+1\right]}-I_{j}^{\left[r+1\right]}|}. \end{array}$$
  Here, *I*_*i*_^[*r*+1]^ is the average intensity of block *i* on level *r* + 1, and $\tilde{\alpha }\geq 0$ is a user-defined parameter. Note that, while the diagonal terms of *A* ^[*r*]^ do not affect the selection of *C*-points on level *r*, they do affect the off-diagonal terms of *A* ^[*r*+1]^ that subsequently influence the selection of *C*-points on level *r* + 1. Diagonal terms of *A* ^[*r*]^ represent the internal similarity of blocks, and should be taken into account when calculating similarity between coarse-level blocks.
- Another blockwise quantity we use to better connect similar blocks is multilevel variance in intensity (as a measure of texture) [21]. Every node on level *r* + 1 corresponds to an overlapping group of nodes on level *r*, every node on level *r* corresponds to an overlapping group of nodes on level *r* − 1, and so forth. The variance in intensity of the level-*r* nodes that correspond to a level-*r* + 1 node is easily calculated using the standard expression var(*X*) = *E*((*X* − *E*(*X*))^2^) = *E*(*X* ^2^) − *E*(*X*)^2^, see (A.12). These variances are calculated between all consecutive levels, and are assigned to coarse-level nodes by recursive averaging (see (A.13)). An *r*-component multilevel feature vector with intensity variances can thus be associated with every node on level *r* + 1, see (A.14). In each such feature vector, the last component gives the variance between the average intensities of the level-*r* nodes that correspond to the level-*r* + 1 node, the one-before-last component gives the average over those level-*r* nodes of the variance between the average intensities of the level-*r* − 1 nodes that correspond to each of those level-*r* nodes, and so forth. The averages in the feature vectors can be calculated efficiently via recursive averaging, see (A.13). The coupling weights between blocks can then be rescaled according to the similarity in these variance vectors, with a scaling parameter *β*, see (A.17). In practice, it turns out that variance for small blocks is less relevant and may cause incorrect segmentation. We therefore only use variance rescaling on levels larger than level *ρ*, with *ρ* a user-defined parameter.
- In order to avoid small segments, we only allow detection of salient segments on levels larger than level *σ*, with *σ* a user-defined parameter.
- In summary, we list the free parameters in our algorithm, to be chosen such that correct segmentation is obtained for the application at hand: top-level intensity scaling factor *α*, coarse-level intensity rescaling factor $\tilde{\alpha }$, coarse-level variance rescaling factor *β*, coarsening strength threshold *θ*, saliency threshold *γ* (see the next section), sharpening threshold *d*_1_, segment detection threshold level *σ*, and variance rescaling threshold level *ρ*.

### 2.3. Scale Invariant Saliency Measure

We propose a saliency measure that is a modified version of the saliency measures used in [8, 9, 12–14, 21]. Our modified saliency measure is scale invariant.

The saliency measures used in [8, 9, 12–14, 21] can be derived and motivated as follows.

On each level *r*, define the energy functional

(7) $$\begin{array}{c} \Gamma^{\left[r\right]}\left(u^{\left[r\right]}\right)=\frac{\sum_{i>j}^{}A_{ij}^{\left[r\right]}{\left(u_{i}^{\left[r\right]}-u_{j}^{\left[r\right]}\right)}^{2}}{\sum_{i>j}^{}A_{ij}^{\left[r\right]}u_{i}^{\left[r\right]}u_{j}^{\left[r\right]}}=\frac{u^{[r]T}L^{\left[r\right]}u^{\left[r\right]}}{\left(1/2\right)u^{[r]T}W^{\left[r\right]}u^{\left[r\right]}}. \end{array}$$

Here, *A* ^[*r*]^ is the coupling matrix on level *r*, the matrices *L* ^[*r*]^ and *W* ^[*r*]^ are given by

(8) $$\begin{array}{c} L_{ij}^{\left[r\right]}=\{\begin{array}{cc} -A_{ij}^{\left[r\right]} & \text{if}\;\;i\neq j,\\ \underset{k\neq i}{\sum }A_{ik}^{\left[r\right]} & \text{if}\;\;i=j, \end{array}\\ W^{\left[r\right]}=A^{\left[r\right]}, \end{array}$$

and *u* ^[*r*]^ is a Boolean state vector for a particular block on level *r* such that *u*_*j*_^[*r*]^ = 1 if node *j* belongs to the block and *u*_*j*_^[*r*]^ = 0 otherwise. Subscripts denote matrix or vector components. *L* ^[*r*]^ is called the Laplacian of the (weighted) graph. Note that *W* ^[*r*]^ = *A* ^[*r*]^ on all levels so there is no real need to introduce the variables *W* ^[*r*]^, but below we choose to use the *W* ^[*r*]^ in expressions for the saliency measure.

 The SWA algorithm seeks segments (Boolean vectors *u* ^[*r*]^) that yield low values of the energy functional. On the finest level, this formulation is equivalent to a normalized cut formulation for the segmentation problem [14, 22]. In the normalized cut approach, a generalized eigenvalue problem is formulated on the finest level that is related to functional (7). The few eigenvectors with lowest eigenvalues are computed and are used to segment the image using a clustering method. Note that AMG solvers can also be developed that directly calculate the eigenvectors of this fine-level eigenvalue problem in an efficient manner [23]. In the SWA approach, however, we do not directly calculate the fine-level eigenvectors, but, instead, consider increasingly coarse versions of the coupling matrix and detect salient segments based on connection strength in those coarse-level coupling matrices, guided by functional (7). This approach allows us to take into account coarse-level features (like multilevel variance) for segment detection.

At level *r*, the SWA algorithm checks for each node *i* whether it is salient. (Recall that node *i* is interpreted as an overlapping block of finest-level pixels.) To this end, define the Boolean vector for the single-node segment with node *i* on level *r* by *u*_*j*_^[*r*],*i*^ = *δ*_*ij*_ (i.e., *u*_*i*_^[*r*],*i*^ = 1, and *u*_*j*_^[*r*],*i*^ = 0 for *j* ≠ *i*). The saliency measure Γ_*i*_^[*r*]^ of node *i* is then given by

(9) $$\begin{array}{c} \Gamma_{i}^{\left[r\right]}=\Gamma \left(u^{[r],i}\right)=\frac{L_{ii}^{\left[r\right]}}{\left(1/2\right)W_{ii}^{\left[r\right]}}, \end{array}$$

and node *i* is designated a salient segment if its saliency measure is smaller than a user-defined constant *γ*:

(10) $$\begin{array}{c} \Gamma_{i}^{\left[r\right]}<\gamma . \end{array}$$

(Note that saliency measure (9) cannot be used on the finest level, since *W*_*ii*_^[1]^ = 0 for all finest-level nodes *i*. In practice, this is normally resolved by not allowing salient nodes on the finest level.) It can be understood easily that saliency measure (9) is sensitive to the scales of segments [8], and, depending on the application, this may lead to difficulties. In what follows, we illustrate the scale sensitivity of saliency measure (9) by a simple example, and propose a new variant of saliency measure (9) that takes into account scaling.

Saliency measure (9) for node *i* on level *r* can be related to the block of pixels on the finest levels that corresponds to node *i* (which we can call block *i*). Indeed, the saliency measure can be interpreted as the sum of the coupling coefficients along the border of block *i* divided by the sum of the coupling coefficients along connections internal to block *i* [12]. It can easily be seen that this interpretation is exact for any nonoverlapping block, as is illustrated by the following simple example.

**Figure 4:**
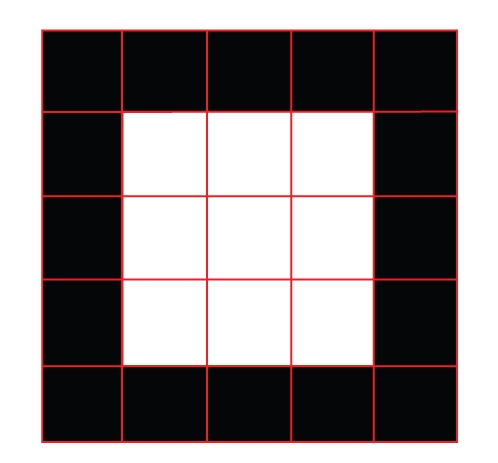
A simple example image with a square block of white pixels in the center. We assume that the white pixels are aggregated to a single, nonoverlapping block on level 2.

Consider Figure 4 which represents an example image with a square block of white pixels in the centre, and assume that this block corresponds to node *i* on level 2 in the aggregation process (we thus call it block *i*). The *i*th column of the interpolation matrix between levels 2 and 1, *P* ^[1,2]^, contains 1 s in the rows corresponding to the pixels in block *i*, and 0 s in the other rows. It is convenient to represent the *i*th column of *P* ^[1,2]^ in so-called stencil form, as

(11) $$\begin{array}{c} P_{i}^{\left[1,2\right]}=\left[\begin{array}{ccccc} 0 & 0 & 0 & 0 & 0\\ 0 & 1 & 1 & 1 & 0\\ 0 & 1 & 1 & 1 & 0\\ 0 & 1 & 1 & 1 & 0\\ 0 & 0 & 0 & 0 & 0 \end{array}\right]. \end{array}$$

Let us define two more matrices, the adjacency matrix *V* ^[1]^ and the unweighted graph Laplacian *G* ^[1]^, which are Boolean versions of *W* ^[1]^ and *L* ^[1]^, not weighted by the coupling strengths in *A* ^[1]^:

(12) $$\begin{array}{c} V_{ij}^{\left[1\right]}=\{\begin{array}{cc} 0 & \text{if}\;\;A_{ij}^{\left[1\right]}=0,\\ 1 & \text{if}\;\;A_{ij}^{\left[1\right]}\neq 0, \end{array}\\ G_{ij}^{\left[1\right]}=\{\begin{array}{cc} -V_{ij}^{\left[1\right]} & \text{if}\;\;i\neq j,\\ \underset{k\neq i}{\sum }V_{ik}^{\left[1\right]} & \text{if}\;\;i=j. \end{array} \end{array}$$

The stencils of the operators *V* ^[1]^ and *G* ^[1]^ are given by

(13) $$\begin{array}{c} V^{\left[1\right]}=\left[\begin{array}{ccc} & 1 & \\ 1 & & 1\\ & 1 & \end{array}\right],\\ G^{\left[1\right]}=\left[\begin{array}{ccc} & -1 & \\ -1 & 4 & -1\\ & -1 & \end{array}\right]. \end{array}$$

(Note that these stencils represent the nonzero elements in each of the columns (or rows) of *V* ^[1]^ and *G* ^[1]^. Since each column has the same pattern of nonzeros, we don not give the stencils of *V* ^[1]^ and *G* ^[1]^ a subscript index. Note that columns corresponding to pixels close to the boundaries of the image would have a different nonzero pattern, but we assume that the white block in Figure 4 is embedded in a larger black image and is located far from the image boundary, such that we don not have to worry about boundaries.)

Let us now calculate the *i*th diagonal elements of the level-2 versions of *V* ^[1]^ and *G* ^[1]^, namely, *V* ^[2]^ = *P* ^[1,2]*T*^ *V* ^[1]^ *P* ^[1,2]^ and *G* ^[2]^ = *P* ^[1,2]*T*^ *G* ^[1]^ *P* ^[1,2]^.

First, we want to show that diagonal element *G*_*ii*_^[2]^ = *P*_*i*_^[1,2]*T*^ *G* ^[1]^ *P*_*i*_^[1,2]^ equals the boundary length of block *i*, which in this example is 3 × 4 = 12. Applying matrix *G* ^[1]^ to column *P*_*i*_^[1,2]^ gives, in stencil notation,

(14) $$\begin{array}{c} G^{\left[1\right]}P_{i}^{\left[1,2\right]}=\left[\begin{array}{ccccc} 0 & -1 & -1 & -1 & 0\\ -1 & 2 & 2 & 2 & -1\\ -1 & 1 & 0 & 1 & -1\\ -1 & 2 & 1 & 2 & -1\\ 0 & -1 & -1 & -1 & 0 \end{array}\right]. \end{array}$$

Multiplying this with row *P*_*i*_^[1,2]*T*^ results in a scalar value of

(15) $$\begin{array}{c} P_{i}^{[1,2]T}G^{\left[1\right]}P_{i}^{\left[1,2\right]}=12, \end{array}$$

which is the boundary length of block *i*, as desired.

Next we want to show that (1/2)*V*_*ii*_^[2]^ = (1/2) × *P*_*i*_^[1,2]*T*^ *V* ^[1]^ *P*_*i*_^[1,2]^ equals the number of internal connections within block *i*. First apply matrix *V* ^[1]^ to column *P*_*i*_^[1,2]^ to get, in stencil notation,

(16) $$\begin{array}{c} V^{\left[1\right]}P_{i}^{\left[1,2\right]}=\left[\begin{array}{ccccc} 0 & 1 & 1 & 1 & 0\\ 1 & 2 & 3 & 2 & 1\\ 1 & 3 & 4 & 3 & 1\\ 1 & 2 & 3 & 2 & 1\\ 0 & 1 & 1 & 1 & 0 \end{array}\right]. \end{array}$$

Then multiply this with row *P*_*i*_^[1,2]*T*^ to obtain

(17) $$\begin{array}{c} P_{i}^{[1,2]T}V^{\left[1\right]}P_{i}^{\left[1,2\right]}=24, \end{array}$$

which equals twice the number of internal connections, or approximately twice the area of block *i*. Due to this interpretation of the diagonal elements of matrices *G* ^[*r*]^ and *V* ^[*r*]^ in terms of block boundary length and block area, we will in what follows refer to the matrices *G* ^[*r*]^ as boundary length matrices, and to *V* ^[*r*]^ as area matrices.

It is now easy to see that the *i*th diagonal element of *L* ^[*r*]^ corresponds to the sum of the coupling coefficients along the boundary of block *i*, or, in other words, to the boundary length of block *i* weighted by the similarity with neighbouring blocks along the block boundary. For this reason, we call the matrices *L* ^[*r*]^ weighted boundary length matrices. Similarly, the *i*th diagonal element of *W* ^[*r*]^ = *A* ^[*r*]^ corresponds to twice the sum of the coupling coefficients along connections internal to block *i*, or, in other words, to twice the area of block *i*, weighted by the mutual similarity of neighbouring finer-level blocks in the interior of block *i*. We therefore call the matrices *W* ^[*r*]^ weighted area matrices. Note that this interpretation also follows directly from plugging *u* ^[*r*]^ = *P*_*i*_^[1,2]^ into (7) (middle expression).

**Figure 5:**
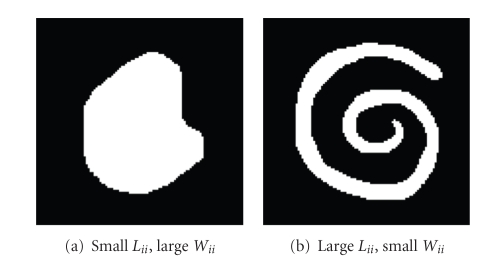
Example shapes for analyzing scale behaviour of saliency measures.

With these interpretations of the diagonal elements of *L* ^[*r*]^, *W* ^[*r*]^, *G* ^[*r*]^ and *V* ^[*r*]^ in hand, we can now analyze the scale behaviour of saliency measure (9), and propose a new scale invariant version of it. Figure 5 shows two examples of images with a white shape on a black background. The original saliency measure

(18) $$\begin{array}{c} \Gamma_{i}^{\left[r\right]}=\frac{L_{ii}^{\left[r\right]}}{\left(1/2\right)W_{ii}^{\left[r\right]}}, \end{array}$$

is much smaller for shape (a) than for shape (b): shape (a) has a shorter boundary and a larger area than shape (b), and the similarity-weighted boundary length *L*_*ii*_^[*r*]^ is proportional to the block boundary length, while the similarity-weighted area (1/2)*W*_*ii*_^[*r*]^ is proportional to the area. For the example considered, this is undesirable, since both shapes should be deemed equally salient: their saliency measure should be of similar value for the SWA algorithm to segment them correctly if they were to occur together in a larger image. It is also clear that saliency measure (18) tends to assume geometrically smaller values as levels get coarser, since, in two dimensions for example, shape areas normally grow by a factor of four when boundary lengths double. This can also be seen as follows. Consider two white blobs with the shape of Figure 5(a), a large one and a small one. The large blob would reduce to a single node at a coarser level than the small blob, and the salience measure (18) for the large blob would be smaller than for the small blob, since the ratio of boundary length to area is smaller for the large blob than for the small blob. We can thus say that saliency measure (18) is not scale invariant (and also not shape-invariant, according to the example of Figure 5). Potential scaling difficulties with saliency measure (18) have been discussed in the literature and some ad hoc fixes have been proposed [8], but a generally applicable modification that addresses these scaling issues has not been proposed yet.

In order to remedy the potential scaling difficulties of saliency measure (18), we propose the following new saliency measure:

(19) $$\begin{array}{c} \Gamma_{i}^{\left[r\right]}=\frac{L_{ii}^{\left[r\right]}/G_{ii}^{\left[r\right]}}{W_{ii}^{\left[r\right]}/V_{ii}^{\left[r\right]}}. \end{array}$$

The motivation behind this new saliency measure is simple: we normalize the similarity-weighted boundary length by dividing it by the unweighted boundary length, and we normalize the similarity-weighted area by dividing it by the unweighted area. As a consequence, the quantities *L*_*ii*_^[*r*]^/*G*_*ii*_^[*r*]^ and *W*_*ii*_^[*r*]^/*V*_*ii*_^[*r*]^ become scale invariant (they lie between 0 and 1, since all coupling weights also lie between 0 and 1). The new saliency measure can then simply be interpreted as

(20) $$\begin{array}{c} \Gamma_{i}^{\left[r\right]}=\frac{\text{average}\;\;\text{similarity}\;\;\text{along}\;\;\text{boundary}\;\;\text{of}\;\;\text{block}\;\;i}{\text{average}\;\;\text{similarity}\;\;\text{in}\;\;\text{interior}\;\;\text{of}\;\;\text{block}\;\;i}. \end{array}$$

This interpretation also provides an easy intuitive understanding of the saliency measure: a block is salient if it has low average similarity to its neighbouring blocks, but this has to be evaluated relative to how similar its own finer-level blocks are to each other. It is also clear that the new saliency measure may make it easier for many applications to choose a saliency threshold *γ* below which nodes are to be considered salient: due to the scale invariance (and shape-invariance) of the new measure the saliency threshold can remain constant on all levels, and segments with different shapes can be detected more consistently.

We have experimented extensively comparing the scale invariant saliency measure (19) and the original saliency measure (18). Even though the scale invariant saliency measure is somewhat more expensive to compute (a second three-way sparse matrix product *V* ^[*r*+1]^ = *P* ^[*r*,*r*+1]*T*^ *V* ^[*r*]^ *P* ^[*r*,*r*+1]^ has to be evaluated on each level), we have found that it is much easier for our application to find a suitable value of the saliency threshold *γ* that works well on all recursive levels and for the different shapes the cells in our images assume. For our application, the extra work leads to a significantly more robust, less parameter-dependent algorithm and is thus worthwhile. It has to be noted, though, that for some applications the original saliency measure (18) may give good results (with less work), or may be preferable for other reasons. The original saliency measure is expected to work well for segments of similar size and shape. Also, the original saliency measure favours large segments (since the saliency measure tends to assume smaller values on coarser levels), and this may be beneficial in applications in which only large segments are of interest. (E.g., one could consider an image with many white blobs of different sizes on a black background, and if only the large blobs are important, the original saliency measure can be used to select them.)

To finalize this section on a scale invariant saliency measure, we want to make three remarks. First, the example in Figure 4 that led to the interpretations of *L*_*ii*_^[*r*]^, *G*_*ii*_^[*r*]^, *W*_*ii*_^[*r*]^ and *V*_*ii*_^[*r*]^ in terms of block boundary lengths and areas, was set in the context of nonoverlapping blocks. In the first stage of the SWA V-cycle, overlapping blocks are employed. The interpretation in terms of block boundaries and areas becomes less straightforward in this case and the heuristics become approximate, but the formulas remain well posed and extensive testing indicates that the measure performs as expected for the case of overlapping blocks as well. Second, saliency measure (19) is not defined on the finest level since *W*_*ii*_^[1]^ = 0 and *V*_*ii*_^[1]^ = 0 for all finest-level nodes *i*. In many applications, salient segments on the finest level are not of interest, and can be disallowed. If they are to be allowed, the areas of finest-level pixels can be set to one, and scale invariant saliency measure Γ_*i*_^[1]^ = *L*_*ii*_^[1]^/*G*_*ii*_^[1]^ can be used. Third, scale invariant saliency measure (19) only uses the diagonal elements of the boundary length and area matrices *L* ^[*r*]^, *G* ^[*r*]^, *W* ^[*r*]^ and *V* ^[*r*]^, and the off-diagonal information in these matrices remains unused. The off-diagonal elements of these matrices can be used to refine (19). For example, if block *i* shares a very short boundary with block *j* to which it is very similar, but is very different from all other neighbouring blocks, then its average similarity along the boundary, *L*_*ii*_^[*r*]^/*G*_*ii*_^[*r*]^, may be very small, even though block *i* is very similar to one of its neighbours. In this case, it may be desirable not to designate block *i* as salient, and to aggregate it with block *j*. Such a situation can be detected by comparing the sizes of *L*_*ij*_^[*r*]^/*G*_*ij*_^[*r*]^ for all neighbours *j* of *i* (these ratios of off-diagonal elements can be interpreted as average similarities between blocks along their mutual boundary), and block *i* may only be considered salient if all of these are small (relative to block *i*'s internal similarity). Improvements of this kind may be important for certain types of applications, and remain a topic of further research.

## 3. Segmentation Results

### 3.1. Cell Segmentation

In this section we evaluate the performance of the multilevel segmentation algorithm by applying it to bright field phase contrast cell microscopy images. First we demonstrate the advantage of segmenting taking the multilevel intensity variance into account, versus not taking it into account.

**Figure 6:**
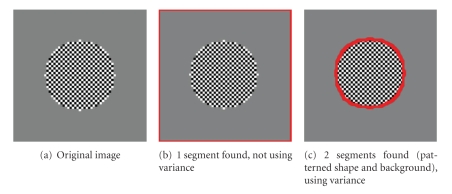
Parameters used: (b) *n* = 60, *α* = 10, $\tilde{\alpha }=10$, *θ* = 0.1, *γ* = 0.1, *d*_1_ = 0.15, *σ* = 5; (c) *n* = 60, *α* = 10, $\tilde{\alpha }=10$, *β* = 10, *θ* = 0.1, *γ* = 0.1, *d*_1_ = 0.15, *σ* = 5, *ρ* = 1.

The image of Figure 6(a) has two regions that differ in texture but are identical in average intensity. Using only intensity, only one segment is found (Figure 6(b)) because the algorithm cannot distinguish the patterned shape from the uniform background. However, when intensity variance is incorporated, the two segments are identified separately (Figure 6(c)). (Note that the SWA algorithm finds the background as a separate segment.)

**Figure 7:**
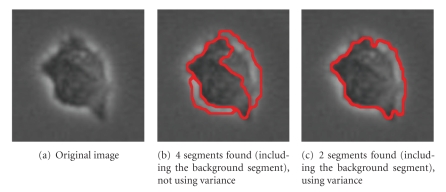
Parameters used: (b) *n* = 60, *α* = 100, $\tilde{\alpha }=10$, *θ* = 0.1, *γ* = 0.5, *d*_1_ = 0.15, *σ* = 6; (c) *n* = 60, *α* = 100, $\tilde{\alpha }=10$, *β* = 30, *θ* = 0.1, *γ* = 0.5, *d*_1_ = 0.15, *σ* = 6, *ρ* = 1.

Next we apply our algorithm to the single isolated cell of Figure 7(a). (All cell images in this and the next section are obtained by cutting out a square *n* × *n* region from larger 512 by 512 images as in Figure 1.) The cell is not segmented correctly when using only intensity. By using variance, however, correct segmentation is obtained: the variance of the background is low, but cells have high internal variance. By preferentially grouping blocks with high variance and blocks with low variance, cell parts are forced to merge together rather than merge with the background.

**Figure 8:**
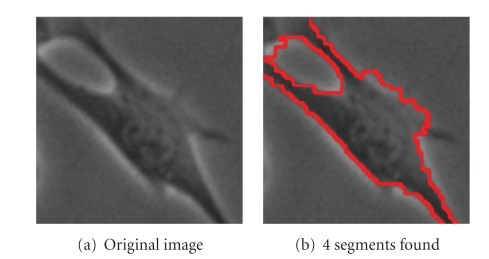
Parameters used: (b) *n* = 60, *α* = 130, $\tilde{\alpha }=5$, *β* = 50, *θ* = 0.08, *γ* = 0.5, *d*_1_ = 0.15, *σ* = 6, *ρ* = 1.

**Figure 9:**
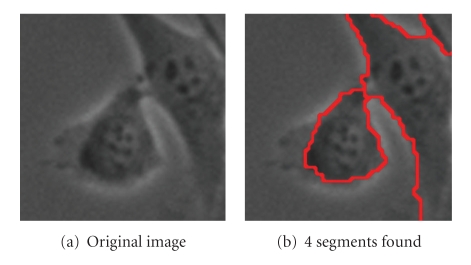
Parameters used: (b) *n* = 60, *α* = 130, $\tilde{\alpha }=4$, *β* = 100, *θ* = 0.1, *γ* = 0.8, *d*_1_ = 0.15, *σ* = 6, *ρ* = 1.

**Figure 10:**
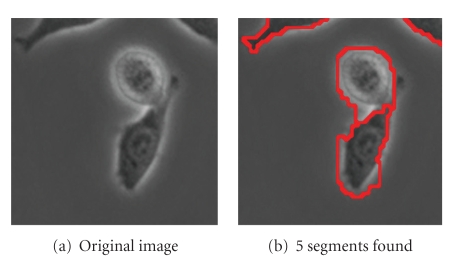
Parameters used: (b) *n* = 60, *α* = 190, $\tilde{\alpha }=5$, *β* = 100, *θ* = 0.09, *γ* = 0.35, *d*_1_ = 0.15, *σ* = 6, *ρ* = 3.

Figures 8, 9 and 10 show further examples of successful segmentation results for increasingly difficult cases of cells with elongated expansions, touching cells, and cells that are close to division (bright and circular). Note that the segmentation parameters employed differ somewhat between the examples; parameter selection is discussed at the end of this section. Also, if the background is not contiguous, the SWA algorithm finds a segment for each of the separate background regions. These background segments can easily be grouped together since they have very similar feature vectors at the level where they are detected as salient segments.

**Figure 11:**
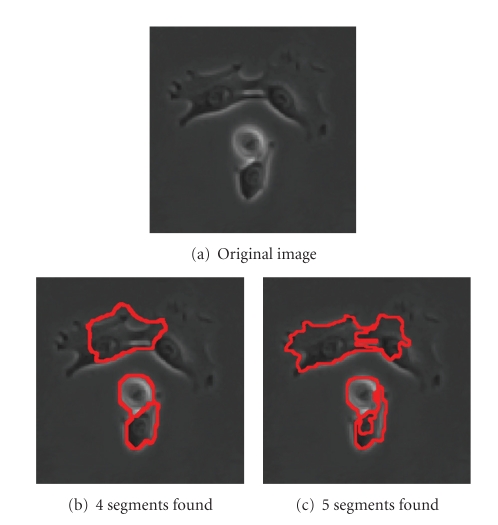
Parameters used: (b) *n* = 120, *α* = 50, $\tilde{\alpha }=0$, *β* = 110, *θ* = 0.15, *γ* = 1.5, *d*_1_ = 0.15, *σ* = 5, *ρ* = 1; (c) *n* = 120, *α* = 100, $\tilde{\alpha }=0$, *β* = 110, *θ* = 0.11, *γ* = 0.9, *d*_1_ = 0.15, *σ* = 5, *ρ* = 3.

Finally, Figure 11 shows segmentation results for a more difficult example involving four whole cells at once, one of which is close to division. With one choice of parameters, we get Figure 11(b), which correctly identifies the 2 cells at the bottom with sharper boundaries but does not segment the top 2 cells correctly. With another choice of parameters, we get Figure 11(c), where all four cells are found. However, part of the top-right cell is segmented with the background, and the nucleus of the bottom cell has its own segment.

### 3.2. Discussion

The results of Figures 7–10 demonstrate that the SWA algorithm is capable of correctly segmenting bright field cell images of moderate complexity. However, some important problems remain. First, it is undesirable that parameters have to be changed depending on the image, since we are pursuing an automatic method. The SWA algorithm contains a number of segmentation parameters that have to be chosen. (The next subsection explains how we have chosen the parameters for our algorithm to obtain the results shown above.) However, the goal of the SWA approach is to include enough multilevel features in the feature vectors of the overlapping blocks on the various levels to allow the algorithm to find correct segments for all images in a certain class, for example, the bright field cell images obtained by our experiment, with a single set of parameters. If this is achieved, the free parameters of the method are not a drawback, but are actually used to finetune the algorithm for the application class at hand, steering the segmentation in the desired direction. In our current implementation we have included multilevel intensity and multilevel intensity variance, and have shown that this allows the algorithm to correctly segment relatively complex bright field cell images. Since these images are difficult to segment, this is a significant achievement, even if somewhat different parameters are required for different images. Note also that the multilevel information about the segments gained by the SWA algorithm leads to several other advantages over commonly used segmentation methods, as discussed in Section 6. However, it is clear that further research is needed before a fully automatic reliable SWA segmentation method is obtained for the bright field images under consideration.

A first type of improvement can be offered by including more features in the feature vector. For example, geometrical shape moments can be calculated for overlapping blocks at all levels, giving information about block shape and orientation that can be used to preferentially group together blocks that have similar shape or orientation [9, 14]. We expect that this can be used to deal better with the difference between cells that are close to division (they appear bright and circular) and regular cells. Anisotropic texture can be considered as well. The algorithm performs well on bright field images containing few cells, but the quality of the segmentation deteriorates when more cells are present. This is mainly due to the low-contrast boundaries and the broken halos that surround the cells. One way to overcome this problem may be to detect and promote boundary integrity across neighbouring aggregates as in [21]. Cross-correlation between features can also be taken into account. Application of these and other algorithmic enhancements will be studied in future research.

A second type of improvement, however, may be achieved by simply considering the extra information that is present in temporal sequences of images. The SWA algorithm can take immediate advantage of this information by applying it directly to these image sequences in space time. However, before exploring SWA segmentation in space time in Section 4, we first discuss how we chose the parameters for the segmentation results of Figures 7–11.

### 3.3. Choice of Segmentation Parameters

To apply the algorithm to an image, we need to choose values for segmentation parameters *α*, $\tilde{\alpha }$, *β*, *θ*, *γ*, *d*_1_, *σ* and *ρ*. In order to find suitable parameters, we normally start with an initial parameter choice, and then finetune individual parameters according to the following guidelines. A suitable initial parameter set for our types of images is given by

(21) $$\begin{array}{c} \left(\alpha ,\tilde{\alpha },\beta ,\theta ,\gamma ,d_{1},\sigma ,\rho \right)=\left(100,100,100,0.1,0.1,0.15,5,1\right). \end{array}$$

1. To increase the contrast level of the image, increase top-level intensity scaling factor *α*, which amplifies the intensity difference between pixel pairs.
2. For images containing broad bright or dark boundaries of regions (such as the halos in the bright field cell images), we do not want these boundaries to become salient segments themselves. Since a large coarse-level intensity rescaling factor $\tilde{\alpha }$ causes blocks with high average intensity to become more disconnected from their neighbours, a bright block can very easily become a salient segment. To avoid this, decrease $\tilde{\alpha }$ to a small value, say in the range [0,5].
3. In order to separate desired segments that differ more in average intensity and less in intensity variance, choose a larger intensity rescaling factor $\tilde{\alpha }$ and a smaller variance rescaling factor *β*. Do the opposite if the desired segments differ more in variance.
4. If the algorithm finds too many (small) segments, try
  - decreasing *θ* (more strong connections),
  - decreasing *γ* (stricter saliency threshold),
  - increasing *σ* (segments allowed only on coarser levels).

On the contrary, if too few (large) segments are found, then shift the parameter values in the opposite direction

(5) The process for segmenting image sequences in space time (see next section) is similar but we usually start with a smaller *θ*, such as 0.03. This is so because a node in an image sequence typically has more neighbours than a node in a single image. If we used the same *θ*, there would be fewer strong connections since our strength criterion defines strength relative to row sums of the coupling matrix (see (A.22)).

## 4. Space Time Segmentation and Tracking

In this section we describe results obtained when applying the multilevel SWA algorithm to sequences of images. In our experimental technique we take images frequently enough that moving cells overlap significantly between frames. (The motion of the cells is slow compared to the image frequency.) Cell trajectories in space time thus form “object tunnels” that are found efficiently by the SWA algorithm. The extra temporal information makes it easier to resolve difficult cases such as touching cells, dividing cells, and cells that temporarily overlap. The resulting space time segments can also be used for tracking cell motion.

By stacking up the images, the problem of segmenting multiple images can be viewed as segmenting one three-dimensional (3D) data set. It is not difficult to modify the SWA algorithm to suit a 3D problem because the algorithm is already designed to coarsen arbitrary graphs, which can represent geometric grids of any dimension. The details of this simple modification are given in Appendix A.4. Additionally, SWA can easily be applied to datasets with three spatial dimensions and one temporal dimension with little or no modicfication.

We now describe segmentation results for sequences of bright field cell images.

**Figure 12:**
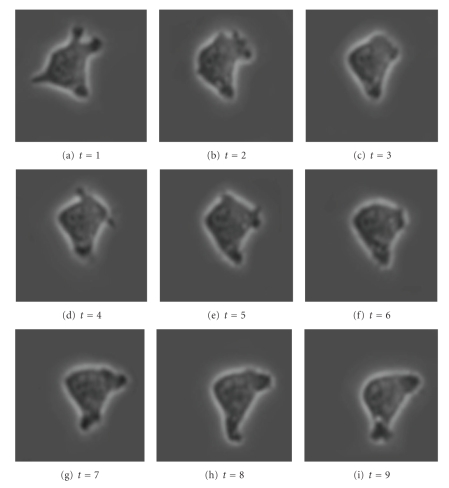
Original image sequence.

**Figure 13:**
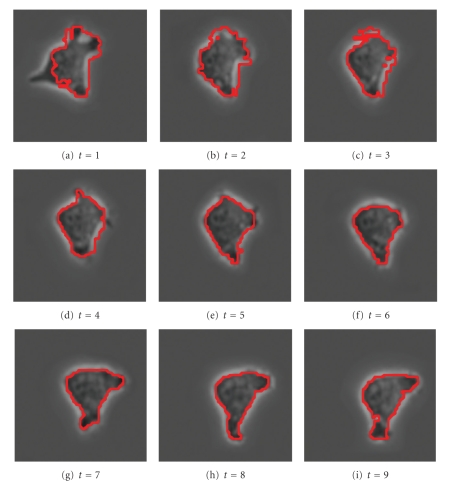
2 segments found with parameters $\left(n,\alpha ,\tilde{\alpha },\beta ,\theta ,\gamma ,d_{1},\sigma ,\rho )=(60,100,5,160,0.0055,0.062,0.15,5,2\right)$.

Figure 12 shows a cell with an irregular shape moving from the top-left corner of the window to the bottom-right corner, while slightly changing its shape, from image *t* = 1 to image *t* = 9. (The actual time between images is 5 minutes.) Applying the SWA algorithm with appropriately chosen parameters to this stack of images, we find the two segments shown in Figure 13. Note that the cell boundary is quite accurately captured in every frame, even though the boundary of the cell is irregular.

**Figure 14:**
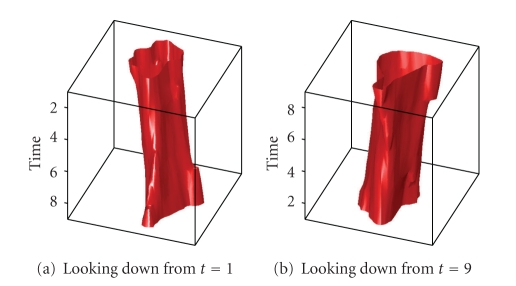
3D representation of the cell segment.

Figure 14 shows a plot of this result in 3D with the red tube representing the cell wall. In Figure 14(a), the images are stacked with the *t* = 1 slice on top. In Figure 14(b), the stack is reversed to show the *t* = 9 slice on top.

**Figure 15:**
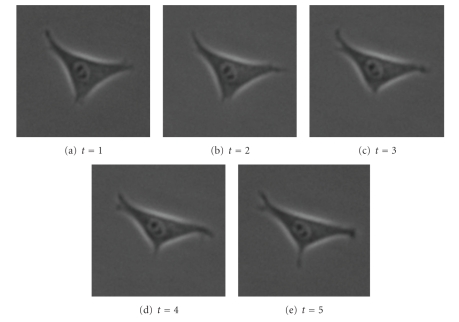
Original image sequence.

**Figure 16:**
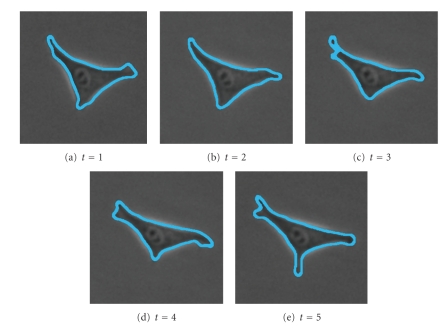
Annotations by a human expert.

**Figure 17:**
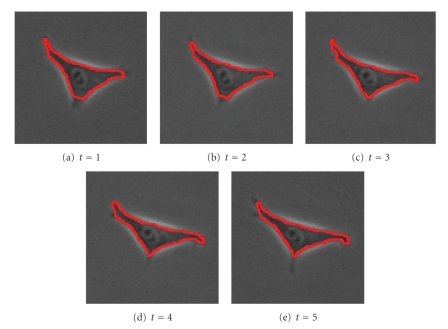
2 segments found with parameters $\left(n,\alpha ,\tilde{\alpha },\beta ,\theta ,\gamma ,d_{1},\sigma ,\rho )=(121,11,30,10,0.025,0.01,0.15,7,3\right)$.

**Figure 18:**
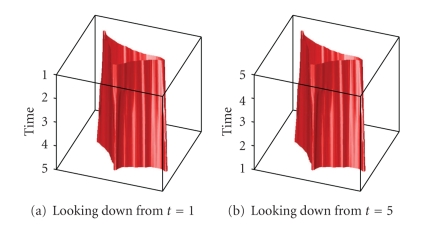
3D representation of the segments.

The next set of images (Figure 15) shows a slow-moving triangular cell, and Figure 16 shows cell contours annotated by a human expert. Figure 17 shows the SWA segmentation results, and Figure 18 gives a 3D representation of them. In comparison with the human expert, the SWA algorithm segments 71634 out of the 121 × 121 × 5 = 73205 pixels correctly, yielding a 97.85% accuracy.

**Figure 19:**
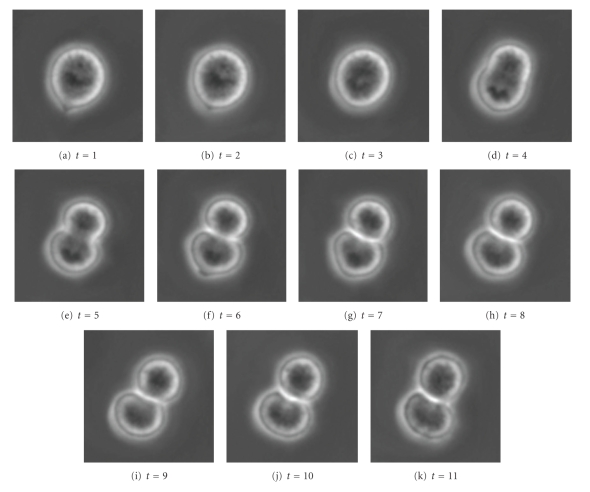
Original image sequence.

**Figure 20:**
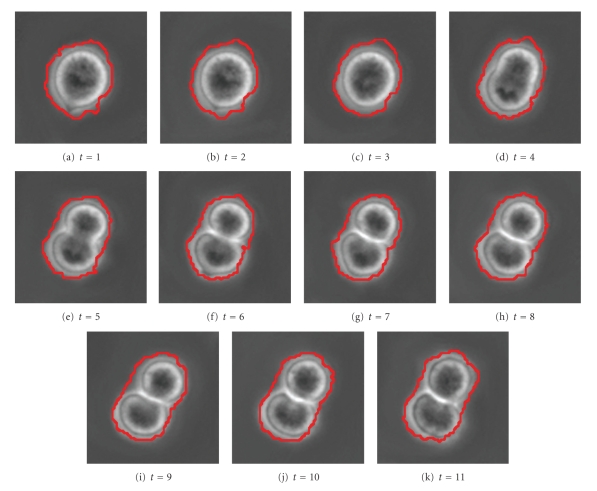
2 segments found with parameters $\left(n,\alpha ,\tilde{\alpha },\beta ,\theta ,\gamma ,d_{1},\sigma ,\rho )=(60,8,4,200,0.025,0.15,0.15,6,1\right)$.

**Figure 21:**
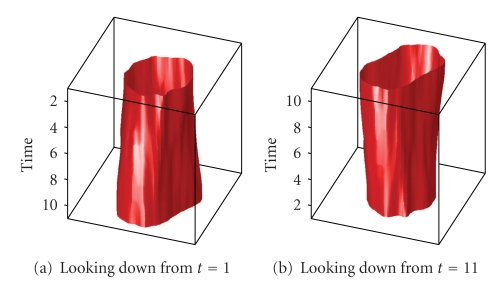
3D representation of the segments.

**Figure 22:**
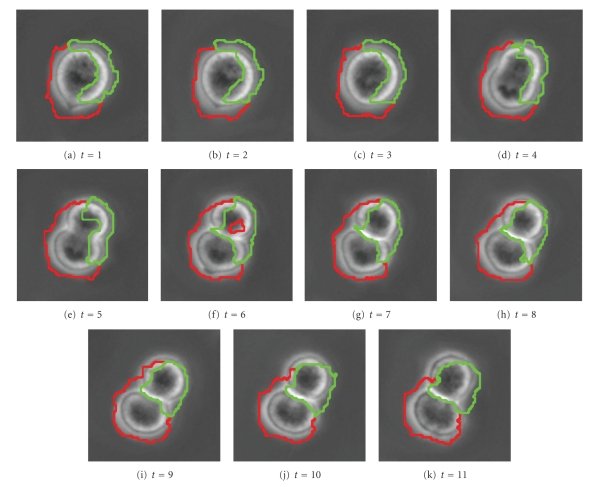
3 segments found with parameters $\left(n,\alpha ,\tilde{\alpha },\beta ,\theta ,\gamma ,d_{1},\sigma ,\rho )=(60,11,2,300,0.015,0.05,0.15,5,4\right)$.

**Figure 23:**
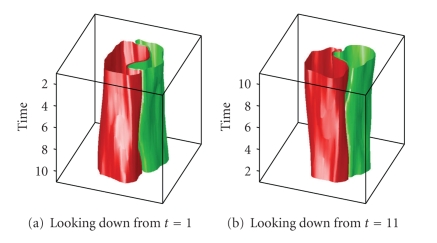
3D representation of the segments.

Figure 19 shows a sequence of 11 cell images which represent a cell dividing into two cells. In Figure 20, segmentation parameters are chosen such that the cells remain in one segment. This may be useful if one desires one large connected segment for all cells in a tree-like structure. Figure 22 shows that segmentation parameters can be modified to obtain two segments, one for each of the daughter cells. Even though the first few images contain only one cell, the information from later times causes the algorithm to interpret the mother cell as two cells that are about to separate. Having two segments instead of one large segment for all three cells may be useful if one desires separate segments for each unique cell. In fact, if earlier images were included, one would want a third segment for the mother cell in this case. These two results illustrate how a judicious choice of segmentation parameters allow the user of the SWA algorithm to steer the segmentation process towards the outcome that is desirable for the application at hand. Figures 21 and 23 show 3D trajectories for the two segmentations. While more research is clearly needed to handle these rather complex cell divisions properly, the preliminary results shown point to interesting possibilities to use the space time SWA algorithm as a building block of comprehensive tracking systems.

## 5. Scalability

Multilevel algorithms often enjoy the desirable properties of fast execution time and low memory cost that are linearly proportional to the number of data elements [9–11]. In this section, we demonstrate how we achieved linear runtime as a function of the number of pixels in the input image for our implementation of the SWA algorithm. We show that this, as expected, also holds for the space time version of the algorithm. This makes the space time SWA algorithm a highly attractive building block for segmentation and tracking systems applied to long sequences of high-resolution images. Also, if applications were to arise in which image sequences with very large numbers of pixels have to be segmented in short time, efficient parallel versions of the SWA algorithm could be developed, along the lines of successful parallel implementations of AMG, which have been shown to scale well on large parallel computers (see, e.g., [24], and references therein).

**Figure 24:**
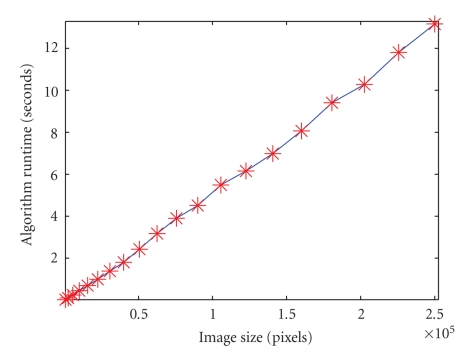
Runtime versus image size. (Single images.)

**Figure 25:**
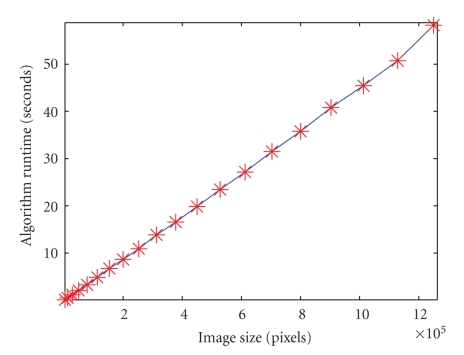
Runtime versus image size. (Space time sequences of images.)

We test runtime scaling by fixing a set of segmentation parameters and running the algorithm on images of different resolution, from 512 × 512 to 10 × 10. (We do this using an original 512 × 512 image that is downsampled to increasingly lower resolution.) For each resolution, we determine the typical runtime by calculating the average runtime over three trials. Figure 24 shows that the runtime scales almost linearly as a function of the number of pixels. Our implementation is a mixed MATLAB-C research code, taking advantage of MATLAB's sparse matrix data structures and operations, and employing C for the compute-intensive parts of the code that cannot easily be done efficiently in MATLAB (in particular, the AMG coarsening step). This implementation could obviously be accelerated significantly for a production environment by migrating the whole implementation to C. However, even in our mixed MATLAB-C implementation, we obtain almost linear scaling, and a quite reasonable runtime of approximately 14 seconds for segmenting a 512 × 512 image. Figure 25 shows that we also obtain linear runtime for a 3D 512 × 512 × 5 image downsampled to increasingly lower resolution.

## 6. Conclusions and Future Work

In this paper, we have investigated the use of a multilevel aggregation algorithm as a method for segmenting live cell bright field microscope images. We use a variant of the Segmentation by Weighted Aggregation (SWA) technique [8, 9] and incorporate an improved scale invariant saliency measure, which can identify salient segments more accurately. We have shown that bright field cell images of moderate complexity are segmented correctly by incorporating multilevel intensity and intensity variance. We have shown how the SWA algorithm can be applied without significant modification to temporal sequences of images, producing segments that track cell contours in space and time. Correct segmentation still depends on a judicious choice of segmentation parameters, and further research is needed before a fully automatic reliable SWA segmentation method is obtained for the bright field images under consideration. In future work, we plan to investigate improving segmentation results by including more features in the feature vector. For example, we are considering to include shape moments, anisotropic texture, cross-correlation between features, and boundary detection [9, 14, 21]. We also plan to apply the multilevel aggregation algorithm to segmentation of cell parts (cytoplasm, nucleus and nucleoli).

While the robust application of multilevel aggregation to bright field cell images requires further research, it is already clear that the SWA segmentation approach may have several advantages over more commonly used segmentation techniques, which include level set, active contour, and watershed methods. First of all, multilevel aggregation is fast and linearly scalable in the number of pixels to be segmented. The algorithm is conceptually simple since there is no need to define initial level set seeds, or extract markers as in watershed. The space time segmentation approach uses extra temporal information that makes it easier to resolve difficult cases like touching cells, dividing cells and cells that temporarily overlap. Nevertheless, it seems that extensive further research is required to handle these difficulties properly. The feature vector for each resulting segment contains multilevel information about the segment that can be exploited in various ways. It can be used to classify segments as background, dividing cell, or regular cell, and it can potentially be used (in 3D) to separate tracks of dividing cells from tracks of regular cells, to aid the proper handling of cell division events. It can also be used to study cell morphology and its dynamics.

The multilevel segmentation approach provides a single, unified technique that produces accurate cell trajectory segments (for moderately complex cases so far) and gives a lot of additional useful information. It is clear that it will have to be combined with other advanced methods of geometric, modeling and statistical nature in order to obtain a complete and robust segmentation and tracking system, along the lines of the sophisticated and comprehensive segmentation and tracking methods that recently have been described (see [5, 6] and references therein). However, due to its conceptual simplicity, linear efficiency, and tunability, and due to the useful additional multilevel information it provides, multilevel aggregation may simplify the development of such comprehensive systems, and it thus promises to be an attractive alternative to level set, active contour and watershed approaches as a basic building block for robust segmentation and tracking systems.

## Appendix

### A. Detailed Description of Multilevel Segmentation Algorithm

#### A.1. Nonrecursive Part

**** Input: an *n* × *n* greyscale image with *N* = *n* ^2^ pixels.

Output: an *N* × *m* Boolean segmentation matrix *U* describing *m* segments (*U*_*i**j*_ = 1 means that pixel *i* belongs to the *j*th segment).

(1) Define global segmentation parameters:
  - *α* (top-level intensity scaling factor),
  - $\tilde{\alpha }$ (coarse-level intensity rescaling factor),
  - *β* (coarse-level variance rescaling factor),
  - *θ* (coarsening strength threshold),
  - *γ* (saliency threshold),
  - *d*_1_ (sharpening threshold),
  - *σ* (segment detection threshold level),
  - *ρ* (variance rescaling threshold level).

(2) Initialize level 1 variables *ℓ* ^[1]^, *M* ^[1]^, *I* ^[1]^, *A* ^[1]^, *S* ^[1]^, *L* ^[1]^, *G* ^[1]^, *V* ^[1]^ and Γ^[1]^:
  - Current level *ℓ* ^[1]^ = 1.
  - Current number of nodes *M* ^[1]^ = *N*.
  - Label the pixels {1,2,…, *M* ^[1]^}. Let *I* ^[1]^ be an *M* ^[1]^ × 1 vector with *I*_*j*_^[1]^ as the intensity of pixel *j*, where the intensity values may range from 0 to 1. (Each input image is scaled such that the maximum intensity value equals 1.)
  - Let coupling matrix *A* ^[1]^ be an *M* ^[1]^ × *M* ^[1]^ matrix with
    (A.1) $$\begin{array}{c} A_{ij}^{\left[1\right]}=\{\begin{array}{cc} e^{-\alpha |I_{i}^{\left[1\right]}-I_{j}^{\left[1\right]}|} & \text{if}\;\;i,j\;\;\text{are}\;\text{horizontal}\;\text{or}\\ & \text{vertical}\;\;\text{neighbours},\\ 0, & \text{otherwise}. \end{array} \end{array}$$
  - Let variance matrix *S* ^[1]^ be an *M* ^[1]^ × *M* ^[1]^ zero matrix.
  - Let weighted boundary length matrix *L* ^[1]^ be an *M* ^[1]^ × *M* ^[1]^ matrix with
    (A.2) $$\begin{array}{c} L_{ij}^{\left[1\right]}=\{\begin{array}{cc} -A_{ij}^{\left[1\right]} & \text{if}\;\;i\neq j,\\ \underset{k\neq i}{\sum }A_{ik}^{\left[1\right]} & \text{if}\;\;i=j. \end{array} \end{array}$$
  - Let area matrix *V* ^[1]^ be an *M* ^[1]^ × *M* ^[1]^ matrix with
    (A.3) $$\begin{array}{c} V_{ij}^{\left[1\right]}=\{\begin{array}{cc} 0 & \text{if}\;\;A_{ij}^{\left[1\right]}=0,\\ 1 & \text{if}\;\;A_{ij}^{\left[1\right]}\neq 0. \end{array} \end{array}$$
  - Let boundary length matrix *G* ^[1]^, be an *M* ^[1]^ × *M* ^[1]^ matrix with
    (A.4) $$\begin{array}{c} G_{ij}^{\left[1\right]}=\{\begin{array}{cc} -V_{ij}^{\left[1\right]} & \text{if}\;\;i\neq j,\\ \underset{k\neq i}{\sum }V_{ik}^{\left[1\right]} & \text{if}\;\;i=j. \end{array} \end{array}$$
  - Let saliency vector Γ^[1]^ be an *M* × 1 vector with
    (A.5) $$\begin{array}{c} \Gamma_{i}^{\left[1\right]}=\frac{L_{ii}^{\left[1\right]}}{G_{ii}^{\left[1\right]}}. \end{array}$$

(3) Determine overlapping segments by calling the recursive graph segmentation function:
  (A.6) $$\begin{array}{cc} U^{\left[1\right]} & =imageVCycle\left(\ell^{\left[1\right]},M^{\left[1\right]},I^{\left[1\right]},A^{\left[1\right]},S^{\left[1\right]},V^{\left[1\right]},\Gamma^{\left[1\right]}\right). \end{array}$$
  (The recursive function *i* *m* *a* *g* *e* *V* *C* *y* *c* *l* *e* is described in Appendix A.2).
(4) Assign pixels uniquely to segments: set
  (A.7) $$\begin{array}{c} U_{ij}⟵\{\begin{array}{cc} 1 & \text{if}\;\;j=\min\;\left\{j:\underset{k}{\max\;}\;U_{ik}^{\left[1\right]}=U_{ij}^{\left[1\right]}\right\},\\ 0, & \text{otherwise}. \end{array} \end{array}$$

#### A.2. Recursive Part (Function *i* *m* *a* *g* *e* *V* *C* *y* *c* *l* *e*)

****Input: *ℓ* ^[*r*]^, *M* ^[*r*]^, *I* ^[*r*]^, *A* ^[*r*]^, *S* ^[*r*]^, *V* ^[*r*]^, Γ^[*r*]^.

Output: *U* ^[*r*]^, in which *U*_*i**j*_^[*r*]^ indicates the fraction of node *i* on level *r* belonging to segment *j*.

(1) If *ℓ* ^[*r*]^ ≤ *σ*, set all Γ_*i*_^[*r*]^ = *∞*. (No salient segments allowed on current level.)
(2) Coarsen the current graph: set
  (A.8) $$\begin{array}{c} C^{\left[r\right]}=coarsenAMG\left(A^{\left[r\right]},\Gamma^{\left[r\right]},\gamma ,\theta \right), \end{array}$$
  where *c* *o* *a* *r* *s* *e* *n* *A* *M* *G* is a function described in Appendix A.3.
(3) Let *M* ^[*r*+1]^ be the length of *C*-point vector *C* ^[*r*]^.
(4) Let *ℓ* ^[*r*+1]^ = *ℓ* ^[*r*]^ + 1.
(5) If *M* ^[*r*]^ = *M* ^[*r*+1]^ (no further coarsening obtained), output *U* ^[*r*]^ as an *M* ^[*r*]^ × *M* ^[*r*]^ identity matrix (every current node is a segment). Otherwise continue.
(6) Let interpolation matrix *P* ^[*r*,*r*+1]^ be an *M* ^[*r*]^ × *M* ^[*r*+1]^ matrix with
  (A.9) $$\begin{array}{c} P_{ij}^{\left[r,r+1\right]}=\{\begin{array}{cc} 1 & \text{if}\;\;i\in C^{\left[r\right]},\;i=C_{j}^{\left[r\right]},\\ 0 & \text{if}\;\;i\in C^{\left[r\right]},\;i\neq C_{j}^{\left[r\right]},\\ \frac{A_{iC_{j}^{\left[r\right]}}}{\sum_{k\in C^{\left[r\right]}}^{}A_{ik}} & \text{if}\;\;i\notin C^{\left[r\right]}. \end{array} \end{array}$$
(7) Let column-scaled interpolation matrix ${\tilde{P}}^{\left[r,r+1\right]}$ be an *M* ^[*r*]^ × *M* ^[*r*+1]^ matrix with
  (A.10) $$\begin{array}{c} {\tilde{P}}_{ij}^{\left[r,r+1\right]}=\frac{P_{ij}^{\left[r,r+1\right]}}{\sum_{k}^{}P_{kj}^{\left[r,r+1\right]}}. \end{array}$$
(8) Let coarse-level intensity vector *I* ^[*r*+1]^ be an *M* ^[*r*+1]^ × 1 vector with
  (A.11) $$\begin{array}{c} I^{\left[r+1\right]}={\left({\tilde{P}}_{ij}^{\left[r,r+1\right]}\right)}^{T}I^{\left[r\right]}. \end{array}$$
(9) For each block on the current coarse level, *r* + 1, compute a new intensity variance measure relative to level *r*:
  (A.12) $$\begin{array}{cc} S_{\text{coarse}}^{\left[r+1\right]} & ={\left({\tilde{P}}^{\left[r,r+1\right]}\right)}^{T}{\left(I^{\left[r\right]}\right)}^{2}-{\left(I^{\left[r+1\right]}\right)}^{2}, \end{array}$$
  and average the previously calculated variance measures for levels finer than level *r* + 1:
  (A.13) $$\begin{array}{cc} S_{\text{fine}}^{\left[r+1\right]} & ={\left({\tilde{P}}^{\left[r,r+1\right]}\right)}^{T}S^{\left[r\right]}. \end{array}$$
  Here, (*I* ^[*r*]^)^2^ and (*I* ^[*r*+1]^)^2^ are the vectors *I* ^[*r*]^ and *I* ^[*r*+1]^ squared componentwise. Then coarse-level intensity variance matrix *S* ^[*r*+1]^ is an *M* ^[*r*+1]^ × (*r* + 1) matrix given by
  (A.14) $$\begin{array}{c} S^{\left[r+1\right]}=\left[\begin{array}{cc} S_{\text{fine}}^{\left[r+1\right]}\;\;|\; & S_{\text{coarse}}^{\left[r+1\right]} \end{array}\right]. \end{array}$$
(10) Define coarse-level coupling matrix *A* ^[*r*+1]^, an *M* ^[*r*+1]^ × *M* ^[*r*+1]^ matrix, in three steps.
  - Let
    (A.15) $$\begin{array}{c} A^{\left[r+1\right]}=P^{[r,r+1]T}A^{\left[r\right]}P^{\left[r,r+1\right]}. \end{array}$$
  - Rescale using coarse-level intensity:
    (A.16) $$\begin{array}{c} A_{ij}^{\left[r+1\right]}⟵A_{ij}^{\left[r+1\right]}e^{-\tilde{\alpha }|I_{i}^{\left[r+1\right]}-I_{j}^{\left[r+1\right]}|}\;\forall i,j. \end{array}$$
  - If *ℓ* ^[*r*+1]^ ≥ *ρ*, rescale using multilevel variance: Set, for all *i*, *j*,
    (A.17) $$\begin{array}{c} A_{ij}^{\left[r+1\right]}⟵A_{ij}^{\left[r+1\right]}e^{-\beta {||s_{i}^{\left[r+1\right]}-s_{j}^{\left[r+1\right]}||}_{2}}, \end{array}$$
    where **s**_*i*_^[*r*+1]^ is the *i*th row of coarse-level intensity variance matrix *S* ^[*r*+1]^.
(11) Let coarse-level weighted area matrix *W* ^[*r*+1]^ = *A* ^[*r*+1]^.
(12) Let coarse-level area matrix *V* ^[*r*+1]^ = *P* ^[*r*,*r*+1]*T*^ *V* ^[*r*]^ *P* ^[*r*,*r*+1]^.
(13) Define coarse-level weighted boundary length matrix *L* ^[*r*+1]^, an *M* ^[*r*+1]^ × *M* ^[*r*+1]^ matrix, in two steps:
  - Let *L* ^[*r*+1]^ = −*A* ^[*r*+1]^.
  - *L*_*i**i*_^[*r*+1]^ ← −∑_*k*≠*i*_ *L*_*i**k*_^[*r*+1]^ for all *i*.
(14) Define coarse-level boundary length matrix *G* ^[*r*+1]^, an *M* ^[*r*+1]^ × *M* ^[*r*+1]^ matrix, in two steps:
  - Let *G* ^[*r*+1]^ = −*V* ^[*r*+1]^.
  - *G*_*i**i*_^[*r*+1]^ ← −∑_*k*≠*i*_ *G*_*i**k*_^[*r*+1]^ for all *i*.
(15) Let coarse-level saliency vector Γ^[*r*+1]^ be an *M* ^[*r*+1]^ × 1 vector, determined as follows. For each *C*-point *i*, if it was salient on level *r*, keep it salient on level *r* + 1. If not, determine its saliency using the saliency measure.

If Γ_*C*_*i*_^[*r*]^_^[*r*]^ = 0, then Γ_*i*_^[*r*+1]^ = 0, otherwise

(A.18) $$\begin{array}{c} \Gamma_{i}^{\left[r+1\right]}=\{\begin{array}{cc} \frac{L_{ii}^{\left[r+1\right]}/G_{ii}^{\left[r+1\right]}}{W_{ii}^{\left[r+1\right]}/V_{ii}^{\left[r+1\right]}} & \text{if}\;\;\frac{L_{ii}^{\left[r+1\right]}\;/G_{ii}^{\left[r+1\right]}}{W_{ii}^{\left[r+1\right]}/V_{ii}^{\left[r+1\right]}}>\gamma \\ 0 & \text{otherwise}. \end{array} \end{array}$$

(16) Recursively segment the coarse graph: Let
  (A.19) $$\begin{array}{cc} U^{\left[r+1\right]} & =imageVCycle(\ell^{\left[r+1\right]},M^{\left[r+1\right]},I^{\left[r+1\right]},A^{\left[r+1\right]},\\ & \;\;\;\;\;\;\;\;S^{\left[r+1\right]},V^{\left[r+1\right]},\Gamma^{\left[r+1\right]}). \end{array}$$
(17) Find the current segmentation matrix from the coarse-level segmentation matrix: Let
  (A.20) $$\begin{array}{c} U^{\left[r\right]}=P^{\left[r,r+1\right]}U^{\left[r+1\right]}. \end{array}$$
(18) Sharpen overlapping segments: For all *i*, *j*, set
  (A.21) $$\begin{array}{c} U_{ij}^{\left[r\right]}⟵\{\begin{array}{cc} 0 & \text{if}\;\;U_{ij}^{\left[r\right]}<d_{1},\\ 1 & \text{if}\;\;U_{ij}^{\left[r\right]}>1-d_{1},\\ U_{ij}^{\left[r\right]} & \text{otherwise} \end{array} \end{array}$$
(19) Return current segmentation matrix *U* ^[*r*]^.

#### A.3. AMG Coarsening (Function *c* *o* *a* *r* *s* *e* *n* *A* *M* *G*)

Input: *A* ^[*r*]^, Γ^[*r*]^, *γ*, *θ*.

Output: *C* ^[*r*]^, indices of *C*-points chosen on level *r*.

1. Let *M* ^[*r*]^ be the number of rows (or columns) in *A* ^[*r*]^.
2. Let *A* ^[*r*]^ be an *M* ^[*r*]^ × *M* ^[*r*]^ matrix containing only strong connections. That is,
  (A.22) $$\begin{array}{c} {\overset{̅}{A}}_{ij}^{\left[r\right]}=\{\begin{array}{cc} A_{ij}^{\left[r\right]} & \text{if}\;\;i\neq j,\;\;A_{ij}^{\left[r\right]}\geq \theta \underset{k\neq i}{\sum }A_{ik}^{\left[r\right]},\\ 0 & \text{otherwise}. \end{array} \end{array}$$
  (One may also use the criterion *A*_*i**j*_^[*r*]^ ≥ *θ* max _*k*≠*i*_ *A*_*i**k*_^[*r*]^ for strong connections, which is more standard in the AMG context. In our experience, either criterion may be used, but *θ* may have to be chosen differently.)
3. Let *λ* be an *M* ^[*r*]^ × 1 vector in which *λ*_*i*_ denotes the number of nonzero entries in column *i* of ${\overset{̅}{A}}^{\left[r\right]}$.
4. Let *T* be an *M* ^[*r*]^ × 1 zero vector that keeps track of the set to which each node is assigned. For node *i*, *T*_*i*_ = 0 means it is unassigned, *T*_*i*_ = 1 means it is a *C*-point, and *T*_*i*_ = 2 means it is an *F*-point.
5. For *i* = 1,2,…, *M* ^[*r*]^, if Γ_*i*_^[*r*]^ < *γ*, then set *T*_*i*_ ← 1 and *λ*_*i*_ ← 0. (So that salient nodes, or segments, are designated as *C*-points.)
6. While not all *T*_*i*_ > 0, do the following.
  - Let *j* be the smallest value satisfying *T*_*j*_ = 0 and *λ*_*j*_ = max _*k*_ *λ*_*k*_. (Nodes that strongly influence many other nodes are likely to be chosen as *C*-points.)
  - Set *T*_*j*_ ← 1 and *λ*_*j*_ ← 0.
  - Let $K=\{k:{\overset{̅}{A}}_{kj}^{\left[r\right]}>0,T_{k}=0\}$. (*K* is the set of unassigned nodes that are strongly influenced by node *j*.)
  - For all *k* ∈ *K*, do the following.
    - Set *T*_*k*_ ← 2 and *λ*_*k*_ ← 0. (Nodes in *K* become *F*-points.)
    - Let $H=\{h:{\overset{̅}{A}}_{kh}^{\left[r\right]}>0,T_{h}=0\}$. (*H* is the set of nodes that strongly influence *k*.)
    - For all *h* ∈ *H*, set *λ*_*h*_ = *λ*_*h*_ + 1. (Nodes in *H* are made more likely to become *C*-points.)
7. Let the vector of *C*-points be *C* ^[*r*]^ = {*i* : *T*_*i*_ = 1}.
8. Return *C* ^[*r*]^.

#### A.4. 3D Modifications

Let *k* be the number of consecutive images we wish to segment in space time. The following modifications need to be made to the nonrecursive part of the algorithm (Appendix A.1). To highlight the changes, we do not rewrite any steps that do not change.

1. Read in the *k* greyscale images as *n* × *n* intensity matrices, *I*_1_^[1]^, *I*_2_^[1]^,…, *I*_*k*_^[1]^. Each input image is scaled such that the maximum intensity value equals 1. Let *N* = *n* ^2^.
2. (The global segmentation parameters are defined as before.)
3. Initialize variables *ℓ* ^[1]^, *M* ^[1]^, *I* ^[1]^, *A* ^[1]^, *S* ^[1]^, *L* ^[1]^, *G* ^[1]^, *W* ^[1]^, *V* ^[1]^ and Γ^[1]^:
  - Set the current number of nodes *M* ^[1]^ = *N* *k*.
  - Obtain the *M* ^[1]^ × 1 intensity vector *I* ^[1]^ by reshaping the *n* × *n* *k* intensity matrix given by
    (A.23) $$\begin{array}{c} \left[\begin{array}{ccccc} I_{1}^{\left[1\right]} & I_{2}^{\left[1\right]} & \cdots & I_{k-1}^{\left[1\right]} & I_{k}^{\left[1\right]} \end{array}\right]. \end{array}$$
  - *A* ^[1]^ is an *M* ^[1]^ × *M* ^[1]^ matrix with
    (A.24) $$\begin{array}{c} A_{ij}^{\left[1\right]}=\{\begin{array}{cc} e^{-\alpha |I_{i}^{\left[1\right]}-I_{j}^{\left[1\right]}|} & \text{if}\;\;i,j\;\;\text{are}\;\;\text{horizontal},\;\;\text{vertical}\\ & \text{or}\;\;\text{time-wise}\;\;\text{neighbours},\\ 0, & \text{otherwise}. \end{array} \end{array}$$
  - The other variables are defined as before.
4. Call the recursive function as before.
5. *U* is defined as before.

Everything else in the algorithm, including the recursive part and the coarsening, remains unchanged.
